# Papillary muscle infarction in relation to left ventricular infarct distribution and transmurality - assessment by delayed enhancement cardiac magnetic resonance imaging

**DOI:** 10.1186/1532-429X-14-S1-P36

**Published:** 2012-02-01

**Authors:** Sean Wilson, Fahmida Islam, Debbie W Chen, Jason Chinitz, Parag Goyal, Kana Fujikura, Thanh Nguyen, Yi Wang, Robert A Levine, Richard B Devereux, Jonathan W Weinsaft

**Affiliations:** 1 Greenberg Cardiology Division/Departments of Medicine Weill Cornell Medical College, New York, NY, USA; 2 Massachussetts General Hospital, Harvard Medical School, Boston, MA, USA; 3 Radiology Weill Cornell Medical College, New York, NY, USA

## Summary

This study used delayed enhancement CMR (DE-CMR) and invasive angiography to evaluate relationships between papillary muscle and left ventricular (LV) chamber wall infarction following ST segment elevation MI (STEMI). Results demonstrate that papillary muscle infarction (PMI) parallels infarct transmurality and contractile dysfunction within the adjacent LV wall.

## Background

Papillary muscles and myocardium within the adjacent LV wall constitute two components of the mitral valve apparatus. Prior studies have demonstrated variable papillary arterial supply, and the relationship between PMI and overall LV infarct pattern is unknown. DE-CMR enables in-vivo study of infarct pattern within the LV - papillary muscle complex.

## Methods

Patients with initial STEMI were enrolled in a prospective imaging registry. CMR (1.5T) was performed within 6 weeks (27±8 days) post-STEMI. Cine-CMR (SSFP) was used to assess LV wall motion (17 segment model, 5 point per-segment score) DE-CMR (IR-GRE, acquired 10-30 minutes post gadolinium [0.2 mmol/kg]) was used to assess infarct morphology: PMI was graded for location and extent (partial or complete, stratified by >50% papillary hyperenhancement); LV infarction was quantified based on global size and regional transmurality (17 segment, 5 point per-segment score). Invasive coronary angiograms were read blinded to CMR.

## Results

**Figure 1:**
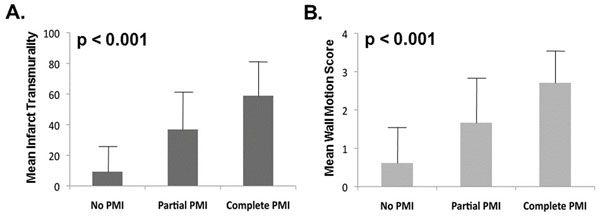
Papillary muscle infarction in relation to left ventricular injury.

153 patients were studied, among whom 30% had PMI (74% posteromedial/37% anterolateral; 11% bilateral). Overall LV infarct size on DE-CMR was larger among patients with PMI (p=0.01). PMI strongly related to LV infarct distribution (Table 1), with prevalence increased 3-fold among patients with lateral wall, and over 1.5-fold with inferior wall infarction on DE-CMR (p≤0.01). Angiography findings paralleled DE-CMR, with over a 2-fold increase in PMI with right coronary artery (RCA) or left circumflex (LCX) culprit vessel infarction (p<0.01). Among patients with RCA infarcts, PMI exclusively occurred (100%) in the setting of right or co-dominant coronary anatomy and was associated with larger angiographic jeopardy score (20.8±6.0 vs. 15.8±5.9, p=0.007). In contrast, only one-third (36%) with PMI and LCX infarcts were left or co-dominant, with similar jeopardy scores between patients with and without PMI (19.4±9.8 vs. 15.3±11.6, p=0.45). Regarding extent, PMI was partial (≤50% hyperenhancement) in 76% of cases. PMI extent paralleled infarct transmurality in adjacent LV segments (Figure 1), with similar results when regional wall motion score was used as a surrogate for LV injury (all p<0.001). Additionally, there was a stepwise increase in LV lateral wall infarct size (% myocardium) among patients with bilateral PMI (12.8±4.2%) compared to those with isolated (3.5±4.2%) or absent PMI (0.8±2.0%) (p<0.001 for trend).

**Table 1:** Infarct size and distribution

	PMI			Posteromedial PMI			Anterolateral PMI		
	
	Present (n=46)	Absent (n=107)	P	Present (n=34)	Absent (n=107)	P	**Present (n=17)** †	Absent (n=107)	P
**INFARCT SIZE**									

**DE-CMR**									
% LV hyperenhancement	19.5±11.4	15.0±9.5	**0.01**	19.2±11.4	15.0±9.5	**0.03**	23.5±14.3	15.0±9.5	**0.002**
**Cardiovascular enzymes**									
Creatine phosphokinase	2590±2344	2164±1836	0.25	2345±1751	2164±1836	0.63	3014±3282	2164±1836	0.34
Creatine phosphokinase-MB	243±207	199±189	0.30	254±202	199±188	0.22	189±212	199±188	0.89
**Duration of symptoms**									
Chest pain interval (hours)	12.4±9.4	10.5±8.4	0.24	12.7±9.7	10.5±8.4	0.24	13.0±9.5	10.5±8.4	0.26

**INFARCT DISTRIBUTION**									

**DE-CMR**									
Anterior wall	35% (16)	70% (75)	**<0.001**	15% (5)	70% (75)	**<0.001**	77% (13)	70% (75)	0.78
Lateral wall	65% (30)	22% (23)	**<0.001**	74% (25)	22% (23)	**<0.001**	59% (10)	22% (23)	**0.003**
Inferior wall	72% (33)	45% (48)	**0.003**	91% (31)	45% (48)	**<0.001**	41% (7)	45% (48)	0.78
**Invasive angiography (infarct related artery)**									
Left anterior descending	28% (13)	72% (77)	**<0.001**	3% (1)	72% (77)	**<0.001**	71% (12)	72% (77)	1.00
Left circumflex artery	24% (11)	6% (6)	**0.001**	32% (11)	6% (6)	**<0.001**	24% (4)	6% (6)	**0.03**
Right coronary artery	48% (22)	22% (24)	**0.002**	65% (22)	22% (24)	**<0.001**	6% (1)	22% (24)	0.19
Anatomically dominant artery*	57% (26)	26% (28)	**<0.001**	77% (26)	26% (28)	**<0.001**	6% (1)	26% (28)	0.12

* Either left circumflex or right coronary artery.

† 5 subjects had concomitant posteromedial and anterolateral PMI.

## Conclusions

PMI is common following STEMI, with PMI extent paralleling infarct transmurality and contractile dysfunction within the adjacent LV wall. Current findings dispute the notion of papillary muscles as end-organ structures particularly susceptible to impaired perfusion, instead supporting the concept that papillary muscles and adjacent LV myocardium are similarly vulnerable to jeopardized arterial supply.

## Funding

K23 HL102249-01, Lantheus Medical Imaging, Doris Duke Clinical Scientist Development Award (Jonathan W. Weinsaft, MD).

